# Rapamycin Protects from Type-I Peritoneal Membrane Failure Inhibiting the Angiogenesis, Lymphangiogenesis, and Endo-MT

**DOI:** 10.1155/2015/989560

**Published:** 2015-11-25

**Authors:** Guadalupe Tirma González-Mateo, Anna Rita Aguirre, Jesús Loureiro, Hugo Abensur, Pilar Sandoval, José Antonio Sánchez-Tomero, Gloria del Peso, José Antonio Jiménez-Heffernan, Vicente Ruiz-Carpio, Rafael Selgas, Manuel López-Cabrera, Abelardo Aguilera, Georgios Liappas

**Affiliations:** ^1^Centro de Biología Molecular-Severo Ochoa (CBMSO), Consejo Superior de Investigaciones Científicas (CSIC), Cantoblanco, 28049 Madrid, Spain; ^2^Departamento de Nefrologia, Hospital das Clínicas da Faculdade de Medicina da Universidade de São Paulo, 05403-000 São Paulo, Brazil; ^3^Aging and Inflammation Group, Instituto de Investigación Biomédica (INIBIC), 15006 A Coruña, Spain; ^4^Departamento de Nefrología, Hospital Universitario de la Princesa, Instituto de Investigación Sanitaria Princesa (IP), 28006 Madrid, Spain; ^5^Departamento de Nefrología, Hospital Universitario La Paz & Instituto de Investigación Sanitaria la Paz (IdiPAZ), 28046 Madrid, Spain; ^6^Departamento de Patología, Hospital Universitario de la Princesa, Instituto de Investigación Sanitaria Princesa (IP), 28006 Madrid, Spain; ^7^Unidad de Biología Molecular y Departamento de Nefrología, Hospital Universitario de la Princesa, Instituto de Investigación Sanitaria Princesa (IP), 28006 Madrid, Spain

## Abstract

Preservation of peritoneal membrane (PM) is essential for long-term survival in peritoneal dialysis (PD). Continuous presence of PD fluids (PDF) in the peritoneal cavity generates chronic inflammation and promotes changes of the PM, such as fibrosis, angiogenesis, and lymphangiogenesis. Mesothelial-to-mesenchymal transition (MMT) and endothelial-to-mesenchymal transition (Endo-MT) seem to play a central role in this pathogenesis. We speculated that Rapamycin, a potent immunosuppressor, could be beneficial by regulating blood and lymphatic vessels proliferation. We demonstrate that mice undergoing a combined PD and Rapamycin treatment (PDF + Rapa group) presented a reduced PM thickness and lower number of submesothelial blood and lymphatic vessels, as well as decreased MMT and Endo-MT, comparing with their counterparts exposed to PD alone (PDF group). Peritoneal water transport in the PDF + Rapa group remained at control level, whereas PD effluent levels of VEGF, TGF-*β*, and TNF-*α* were lower than in the PDF group. Moreover, the treatment of mesothelial cells with Rapamycin* in vitro* significantly decreased VEGF synthesis and selectively inhibited the VEGF-C and VEGF-D release when compared with control cells. Thus, Rapamycin has a protective effect on PM in PD through an antifibrotic and antiproliferative effect on blood and lymphatic vessels. Moreover, it inhibits Endo-MT and, at least partially, MMT.

## 1. Introduction

Peritoneal dialysis (PD) is a form of renal replacement therapy based on the ability of the peritoneal membrane (PM) to perform diffusive and convective transport, in order to maintain solute and fluid equilibrium in uremic patients. Ultrafiltration (UF) failure and the consequent extracellular volume overload are one of the major causes of PD abandonment [1]. In early stages, membrane damage may manifest itself as an increase in water and solute transport, but, as the lesion progresses, transport decreases and underdialysis may take place. The main factors involved in UF failure are peritoneal chronic and acute inflammation, which may lead to progressive deterioration of PM function (type-I PM failure).

Glucose degradation products (GDPs), formation of advanced glycation end-products (AGEs), uremic toxins, and low pH in PD fluids (PDF) play important roles in PM deterioration [2]. These bioincompatible features of PDFs induce an immunological response in PM, involving mesothelial cells (MCs), macrophages, lymphocytes, and neutrophils. They are stimulated to produce a variety of cytokines and growth factors, such as tumor necrosis factor- (TNF-) *α*, interleukin- (IL-) 1, IL-8, transforming growth factor- (TGF-) *β*, vascular endothelial growth factor (VEGF), and fibroblast growth factor- (FGF-) 2, which amplify the inflammation, with structural and functional consequences [3]. One important consequence of PDFs bioincompatibility is the induction of MCs transdifferentiation, a phenomenon known as mesothelial-to-mesenchymal transition (MMT) [4]. Transdifferentiated cells have been detected in PM even before the onset of fibrosis [5], a second consequence of PD. They are also major contributors to VEGF and TGF-*β* production in the peritoneum, providing more stimuli for extracellular matrix (ECM) production (fibrosis) and enhancement of the local vascular networks [6], leading to angiogenesis, a third consequence of PD. The result is a thickened and hypervascularized PM, which leads to the changes in fluid and solute transport observed in patients in the long term of PD [5, 6]. Another important process involved in peritoneal pathogenesis is the endothelial-mesenchymal transdifferentiation (Endo-MT). Its importance has been recently recognized and between 3 to 5% of submesothelial PD fibroblasts seem to derive from this process [7]. MMT and Endo-MT share common triggering mechanisms. Both begin with TGF-*β* overproduction and activation of Smads cascade, leading to VEGF hyperproduction [8, 9]. But VEGF production is not only regulated by TGF-*β*; glucose from PDFs* per se* also induces VEGF upregulation, as well as vascular hyperpermeability and PD dysfunction, as has been demonstrated [10]. The implication of VEGF in both, early and late stages of PM failure (inducing high vascular permeability and hypervascularization, resp.), leads us to hypothesize that VEGF may be considered a therapeutic target. In addition, VEGF hyperproduction leads to another of the consequences of PD: peritoneal lymphangiogenesis, which is mainly regulated by VEGF-C and VEGF-D and is closely linked to MMT [6, 11]. Although poorly studied, lymphangiogenesis is one of the most decisive factor implied in peritoneal water transport disorders [1].

Rapamycin is a macrolide antibiotic produced by* Streptomyces hygroscopicus* that shows antifungal, immunosuppressant, and antitumor properties. Its therapeutic effects are derived from its ability to inhibit the so-called mammalian target of rapamycin (mTOR) complex and include antifibrotic, cytostatic, antiangiogenic, and anti-lymphangiogenic effects [12] as has been demonstrated in tumors, especially carcinomas and hematologic cancers, as well as in retinal and corneal diseases [13–15]. The same anti-VEGF effect could also explain the inhibition of lymphangiogenesis showed by Rapamycin in kidneys [16]. We wondered if this drug could have an effect over Endo-MT, which may be involved in tissue fibrosis.

Recently, two articles analyzed the antifibrotic effect of Rapamycin on the PM in a rat PD model [17, 18]. Xu et al. focused their discussion on the capacity of Rapamycin to inhibit the extracellular matrix production and to improve the peritoneal transport [17]. Ceri et al. [18] studied the regression of peritoneal sclerosis in a rat model of encapsulating peritoneal sclerosis (EPS) treated with Rapamycin. Unfortunately, none of them analyzed the intraperitoneal antilymphangiogenic effect of this drug.

According to the aforementioned data, we suggest that the PM deterioration might be treated with Rapamycin, given its pleotropic effects including antiangiogenic, antilymphangiogenic, antifibrotic, and anti-inflammatory properties. The goal of this study was to analyze the effects of Rapamycin on PM damage in a mice PD model. We also analyzed its effect on MCs viability, cytokine production, and wound healing capacity. Herein, we demonstrated that Rapamycin protects the PM in PD, modulating the tissue fibrosis, MMT, Endo-MT, and especially angiogenesis and lymphangiogenesis.

## 2. Materials and Methods

### 2.1. Peritoneal Dialysis Fluid Exposure Model in Mice

A total of 21 female C57BL/6 mice aged between 12 and 16 weeks were used in this study (Harlan Interfauna Iberica, Barcelona, Spain). The experimental protocol used was in accordance with the National Institutes of Health Guide for Care and Use of Laboratory Animals and was approved by the Animal Ethics Committee of the “Unidad de Cirugía Experimental” of “Hospital Universitario la Paz.” Food and water were provided ad libitum to the animals.

PDF or saline was instilled via a peritoneal catheter connected to a subcutaneous mini access port (Access Technologies, Skokie, IL, USA) as previously described [7, 19]. During the first week after surgery, the animals received 0.2 mL of saline with 1 IU/mL heparin. Thereafter, during a 4-week period, 6 mice did not receive any treatment (control group), 7 mice were daily instilled with 2 mL of standard PD fluid (PDF group) composed of 4.25% glucose and buffered with lactate (Stay Safe; Fresenius, Bad Homburg, Germany), and 7 mice were treated with oral Rapamycin diluted in water (2 mg/kg/day in 15 *μ*L of volume administered to mice using a pipette and a gastric tube) and daily instilled with 2 mL of standard PD fluid (PDF + Rapamycin group).

Two animals of the PDF group and one from PDF + Rapamycin group were not used in the final analysis, because of catheter port infections related to skin wounds (control group, *n* = 6; PDF group, *n* = 5; and PDF + Rapamycin group, *n* = 6). A peritoneal equilibrium test was performed in the last day of experiment. For this purpose, all mice were instilled with 2 mL of PD solution (dextrose 4.25%). After 30 min, they were anaesthetized with isoflurane (MTC Pharmaceuticals, Cambridge, ON, Canada) and sacrificed, so that all the peritoneal fluid volume that remained in the cavity could be recovered, as previously described [7, 19, 20]. Briefly, this method consists of opening the peritoneal cavity through an incision in the muscle and extracting all the fluid with a pipette. There is always a minimum percentage of remaining volume that is not possible to extract.

Diaphragmatic and parietal peritoneum samples were obtained from the contralateral side of the implanted catheter.

### 2.2. Histological Analyses of Peritoneal Samples and Effluent Growth Factors Measurements

For histological analysis, parietal peritoneum from the opposite side to the catheter insertion site was divided into several pieces, avoiding the* linea alba*. One piece was directly frozen in OCT (optimal cutting temperature) compound for immunofluorescence analysis and two other pieces were routinely fixed in Bouin or neutral buffered 3.7% formalin and embedded in paraffin to obtain 5 *μ*m tissue sections.

Deparaffinized sections from Bouin fixed samples were stained with Masson's trichrome. The submesothelial thickness was determined in a blinded manner, by microscope analysis and the mean of independent measures every 60 *μ*m was considered for each animal.

For immunohistochemical studies, diaphragmatic and peritoneal tissue samples previously fixed in neutral buffered 3.7% formalin were cut into 3 *μ*m sections and heated to expose the hidden antigens using Real Target Retrieval Solution containing citrate buffer, pH 6.0 (Dako, Glostrup, Denmark). Samples were also pretreated with Real Peroxidase-Blocking Solution (Dako) to block endogenous peroxidase. A biotinylated goat IgG (H + L) (Vector Laboratories, Burlingame, CA, USA) was applied to detect primary antibodies CD31 (Abcam, Cambridge, UK) and podoplanin (PA2.26, a gift from Dr. Gamallo, Laboratory of Pathology, Hospital de La Princesa, Madrid, Spain). Complexes were visualized by R.T.U Vectastain Elite ABC Kit (Vector Laboratories) and using DAB (Dako) as chromogen. Finally, all cases were counterstained with haematoxylin. In order to estimate the vessel density, 6 arbitrary 20x fields in the submesothelial peritoneal area were analyzed and quantified using the scale bar method (numbers of CD31^+^ cells/*μ*m^2^). Images were analyzed by computerized digital image analysis (AnalySIS, Soft Imaging System). Number of cells with single or double positive staining was counted and was expressed as the mean of 10 independent measurements for each animal. Podoplanin positive lymphatic vessels in the diaphragms of 6 arbitrary 10x fields for each animal were quantified using the analysis program Image-J 1.37c (National Institute of Health, Bethesda, Maryland).

For immunofluorescence analysis, cryostat sections (5 *μ*m) from frozen samples were stained with antibodies to visualize vasculature (CD31; Serotec, Oxford, UK), MCs (Pan-Cytokeratin; Sigma-Aldrich), and pathologic fibroblasts (FSP1; Dako). The sections were fixed for 15 minutes in 4% formaldehyde in PBS and blocked with 10% horse serum for 1 hour in PBS with 0.3% Triton X-100. First antibodies were incubated in PBS with 0.1% Triton X-100 overnight at 4°C. After 3 washing steps, secondary Alexa-labelled antibodies were incubated for 90 minutes at room temperature. After another washing process, the preparations were mounted with a 4,6-diamidino-2-phenylindole (DAPI) nuclear stain (Vectashield; Vector Laboratories). Negative controls for immunofluorescence staining were conducted using 10% rabbit serum instead of primary antibody. The cytokeratin and FSP-1 costaining defined the MMT and the CD31 and FSP1 costaining defined the Endo-MT.

The amounts of VEGF-A, TGF*β*1, and TNF-*α* in peritoneal effluent were obtained the last day of experiment (PET day) and determined by ELISA-based assays, according to manufacturers' instructions (BMS619, Bender MedSystems, Vienne, Austria; DY1679, R&D Systems, Minneapolis, USA; and 560478, BD Company, BS, San José, CA, USA, resp.).

### 2.3. Differences in Peritoneal UF Rate among Mice Exposed to PD versus Controls

In order to analyze the changes in water peritoneal transport between peritoneum exposed to PD and virgin peritoneums, a separate study was performed: twenty mice with virgin peritoneum (control group) received an intraperitoneal injection of 2 mL of Stay Safe 4.25% (Fresenius Medical Care). These mice were sacrificed extracting the total water volume from peritoneal cavity at 20, 30, 40, and 60 minutes after intraperitoneal injection (UF test). Another twenty mice were subjected to PD receiving and intraperitoneal infusion of Stay Safe 4.25%, 2 mL per day, during 30 days. At the end of the experiment we performed the same UF test. Five mice were analyzed in each time point (20, 30, 40, and 60 min) for both groups.

### 2.4. Culture of Omentum and Effluent-Derived MCs and Treatments

MCs were obtained from omental samples of patients undergoing elective abdominal surgery and from peritoneal effluents of PD patients as previously described [21]. These cells were cultured in Earle's M199 medium supplemented with 20% fetal calf serum, 50 U/mL penicillin, 50 *μ*g/mL streptomycin, and 2% Biogro-2 (containing insulin, transferrin, ethanolamine, and putrescine: Biological Industries, Beit Haemek, Israel). The purity of omentum- and effluent-derived MCs cultures was determined by the expression of standard mesothelial markers: intercellular adhesion molecule- (ICAM-) 1, calretinin, and cytokeratins. These MCs cultures were negative for von-Willebrand factor and CD45, ruling out any contamination by endothelial cells or macrophages [21]. To induce MMT* in vitro*, omentum-derived MCs were initially seeded in bottle flash (25 or 100 mL) and then plated at P6 (50.000 cells/mL in M199 medium, Bauer Chamber). When they reached subconfluence, MCs seeded on wells coated with collagen I (50 *μ*g/mL, Roche Diagnostics GmbH, Mannheim, Germany) and treated in different time points (6 to 48 hours) with human-recombinant TGF-*β*1 (1 ng/mL, R&D Systems Inc., Minneapolis, MN, USA), a commonly used* in vitro* model of MMT [21]. Where indicated, Rapamycin (Wyeth Laboratories, Madison, NJ, USA) was administered at concentrations of 2 and 4 nM. Effluent-derived MCs that had undergone MMT (as determined by nonepithelioid morphology, by low expression of E-cadherin, and by upregulated expression of mesenchymal markers) were also treated with different doses of Rapamycin (2, 4, and 20 nM) and analyzed at 6, 24, and 48 hours. In order to establish a correction factor we measured the total protein in each well at the end of the experiment.

The present study adjusts to the Declaration of Helsinki and it was approved by the Ethics Committee of “Hospital Universitario de la Princesa” (Madrid, Spain). Informed written consent to use effluent samples was obtained from all the PD patients included in this study and oral informed consent was obtained from omentum donors subjected to elective surgeries.

### 2.5. Western Blot, Quantitative RT-PCR, and Enzyme-Linked Immunoassays

For western blotting, MCs cultures were lysed in a buffer containing 1% sodium deoxycholate and 0.1% sodium dodecyl sulfate (SDS). Total protein was quantified using a protein assay kit (Bio-Rad, Hercules, CA). Total cell protein (50 *μ*g) was resolved on 8–10% SDS-polyacrylamide gels and transferred to nitrocellulose membranes, which were then blocked with fat-free milk and probed with specific antibodies against E-cadherin, *α*-SMA, collagen I, fibronectin, and tubulin (Sigma-Aldrich, Inc., St. Louis, MO). These antibodies were detected with peroxidase conjugated goat anti-mouse IgG antibody (BD Biosciences, Franklin Lakes, NJ) and visualized by enhanced chemiluminescence (ECL detection kit, Amersham Biosciences, Freiburg, Germany). Images of the blots were acquired with an LAS-1000 Charge Coupled Device camera (Fujifilm, Cedex, France).

For quantitative RT-PCR analysis, MCs were lysed in TRI Reagent (Ambion Inc., Austin, TX), and RNA was extracted as fabricant instructions. Complementary-DNA was synthesized from 2 *μ*g of total RNA by reverse transcription (RNA PCR Core Kit, Applied Biosystems Inc., New Jersey). Quantitative PCR was carried out in a Light Cycler 2.0 using a SYBR Green Kit (Roche Diagnostics GmbH) and specific primers sets for Snail, E-cadherin, and histone H3. Samples were normalized with respect to the value obtained for H3. The primer sets employed for Snail, E-cadherin, and H3 have been previously described [7].

VEGF-A, VEGF-C, and VEGF-D were measured in the culture supernatants by ELISA kits (R&D Systems Inc., USA): VEGF-A, catalog number DVE00 and sensitivity 9 pg/mL; VEGF-C, catalog number DVEC00 and sensitivity 48.4 pg/mL; VEGF-D, catalog number DVED00 and sensitivity 31.3 pg/mL. For all of them, the cross-reactivity observed with available related molecules was <0.5%.

### 2.6. Proliferation Assays

For proliferation assays, 10^4^ cells/well of omentum derived MCs with epithelioid phenotype (semiconfluent) were seeded into 96-well plates and cultured at 37°C, 5% CO_2_ and incubated with Rapamycin 2, 4, and 20 nM for 48 hours. Cells were then pulsed with [3H]-thymidine (1 mCi per well) for 16–18 hours and lysed with Filter Mate Cell Harvester (Perkin Elmer, Turku, Finland). Radioactivity was determined for 1 minute in a basic beta liquid scintillation counter (Perkin Elmer).

As a complementary cell proliferation test and using the same experimental conditions as the previous assay, we also measured total protein synthesis (*μ*g/mL) using Pierce, BCA protein assay kit (Life Technologies, USA).

### 2.7. Cell Cycle and Wound Healing

To analyze the effect of Rapamycin on cell cycle profile, omentum MCs were grown in fetal calf serum and treated with different doses of Rapamycin (2, 4, and 20 nM) for 48 h. Cells were trypsinized, pelleted, and fixed with 70% cold ethanol for 30 min. After washing, samples were suspended in PBS and an equal volume of propidium iodide solution, containing 200 *μ*g/mL RNase (Sigma Aldrich), 20 *μ*g/mL propidium iodide (Sigma Aldrich), and 0.1% Triton X-100 in PBS, was added to the cell suspension for 30 min at room temperature. A FACS Calibur flow cytometer (BD Bioscience) was used to analyze DNA content; emitted light was measured at 675 nm.

To test the effect of Rapamycin on MCs wound repair capacity, a wound healing experiment was performed with cells treated or not with different doses of Rapamycin. Briefly, MCs from omentum, treated or not with Rapamycin, were subjected to mechanical injury with an adapted cell scraper approximately 1500 *μ*m in width and photographed every eight hours during 72 hours. MCs treated with high doses of Rapamycin showed a slight delay at 24 and 48 hours in wound closure.

### 2.8. Statistical Analysis

**Figure 1:**
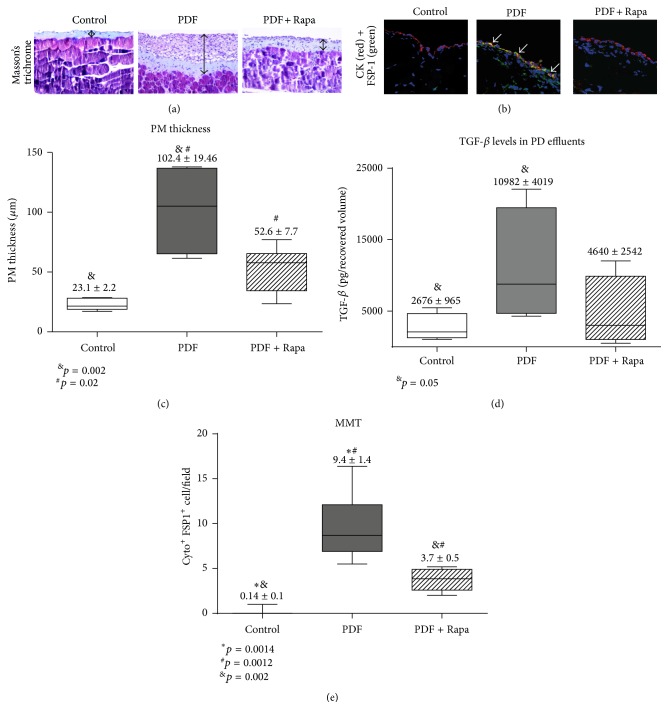
Rapamycin decreases the PM deterioration in a PD mouse model. Mice received a daily instillation of standard PD fluid (2 mL per day) through a intraperitoneal catheter during 4 weeks with or without the oral administration of Rapamycin (2 mg/kg/day: PDF, *n* = 5, and PDF + Rapamycin, *n* = 6). A control group of mice that were only exposed to the presence of the catheter was also included (control; *n* = 6). Peritoneal samples were prepared and analyzed as described in Materials and Methods section. (a) Standard PD fluid exposure increases matrix deposition (blue stained zones) and the thickness of the PM (black lines), while Rapamycin administration significantly reduces these effects when measured in Masson's trichrome staining (sections representative slides). Magnification 200x. (b) As shown in immunofluorescence staining of cytokeratin (red) and FSP-1 (green) (counterstained with DAPI in blue), PDF exposure-promoted MMT (cells coexpressing cytokeratin and FSP-1 with double positive staining, yellow) is reduced by Rapamycin administration. Magnification 400x. (c) The peritoneal thickness (*μ*m) is increased in PDF group compared with control mice, and the group PDF with Rapamycin shows a significant reduction of thickness when compared with PDF group. Analysis of variance results in a significance of *p* = 0.001 (one-way ANOVA test). (d) Measurement of TGF-*β*1 (pg/mL) in the drained volumes shows a gentle increase (although not statistically significant) of this growth factor in PD fluid-instilled animals while Rapamycin administration tends to reduce TGF-*β*1 production. The analysis of variance results in a *p* value of 0.146 (one-way ANOVA test). (e) Numbers of mesothelial cells per field suffering MMT increase during PDF exposition, while Rapamycin is able to reduce the occurrence of this pathological process. The analysis of variance results in a significance of *p* < 0.0001 (one-way ANOVA test). Box plots graphics represent the median, minimum, and maximum values, as well as the 25th and 75th percentiles. Numbers above boxes depict means ± SE. Symbols represent the statistical differences between groups analyzed by Mann-Whitney *U* test.

**Figure 2:**
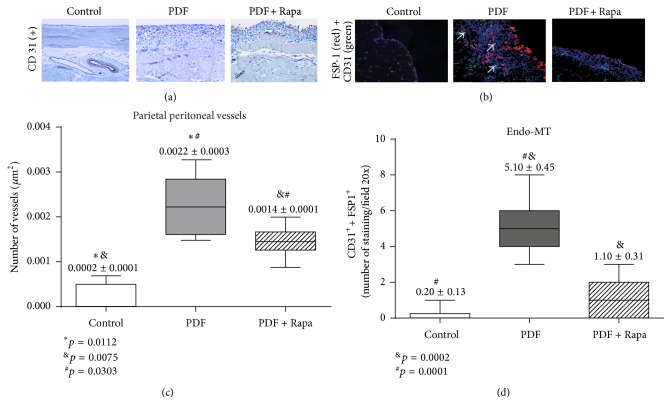
Treatment with Rapamycin decreases PD-induced angiogenesis and Endo-MT. Mice received a daily instillation of standard PD fluid with or without the oral administration of Rapamycin (PDF; *n* = 5 and PDF + Rapamycin; *n* = 6). A control group of mice was also included (control; *n* = 6). (a) Standard PD fluid exposure increases peritoneal angiogenesis and Rapamycin administration significantly reduces the number of submesothelial blood vessels, as determined by CD31 staining (number of CD31^+^ cells/*μ*m^2^). Magnification 200x. (b) Standard PD fluid exposure also increases the presence of endo-MMT, measured as double positive immunofluorescence staining (white arrows) of CD31 (green) and FSP-1 (red) (counterstained with DAPI, in blue), which is reduced by Rapamycin administration. Magnification 200x. (c) Box plots represent the number of submesothelial CD31^+^ vessels stained cells per field in the different experimental groups and show a decrease of angiogenesis in the Rapamycin-treated animals. The analysis of variance results in a significance of *p* < 0.0001 (one-way ANOVA test). (d) The numbers of double stained CD31^+^/FSP-1^+^ cells per field increase in the PDF-exposed group and show a decrease in the Rapamycin-treated animals. The one-way ANOVA test resulted in a significance of *p* < 0.0001. Box plots graphics represent 25th and 75th percentiles and median, minimum, and maximum values. Numbers above boxes depict means ± SE. Symbols represent the statistic differences between groups.

**Figure 3:**
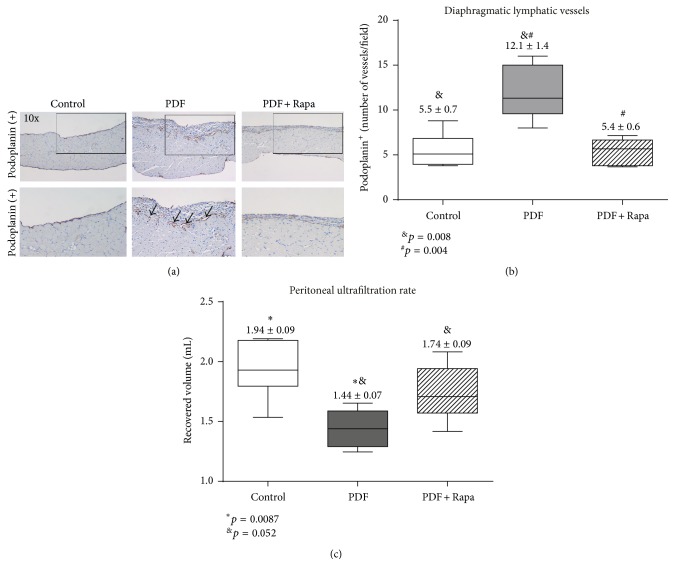
Treatment with Rapamycin diminished lymphangiogenesis, improving peritoneal ultrafiltration rate. (a) The diaphragm was stained with podoplanin to analyze the side of the peritoneal cavity. Immunohistochemical staining of podoplanin reveals an increase of submesothelial lymphatic vessels during PDF exposition, while this lymphangiogenesis is reduced with Rapamycin. Images were taken with a 10x objective. Insets below show a detail of the part selected with a square in each picture. (b) Submesothelial count of lymphatic vessels (podoplanin positive staining). Rapamycin showed significantly lower number of lymphatic vessels than the PDF group and similar to controls (*p* = 0.0002, one-way ANOVA test). (c) A 30-minute UF test was performed on the last day of treatments. The volumes recovered from animals exposed to PD fluid are lower than those from control mice and an increase of net UF is obtained in mice exposed to PD fluid that were administrated Rapamycin. A significance of *p* = 0.0064 was obtained with the analysis of variance test.

**Figure 4:**
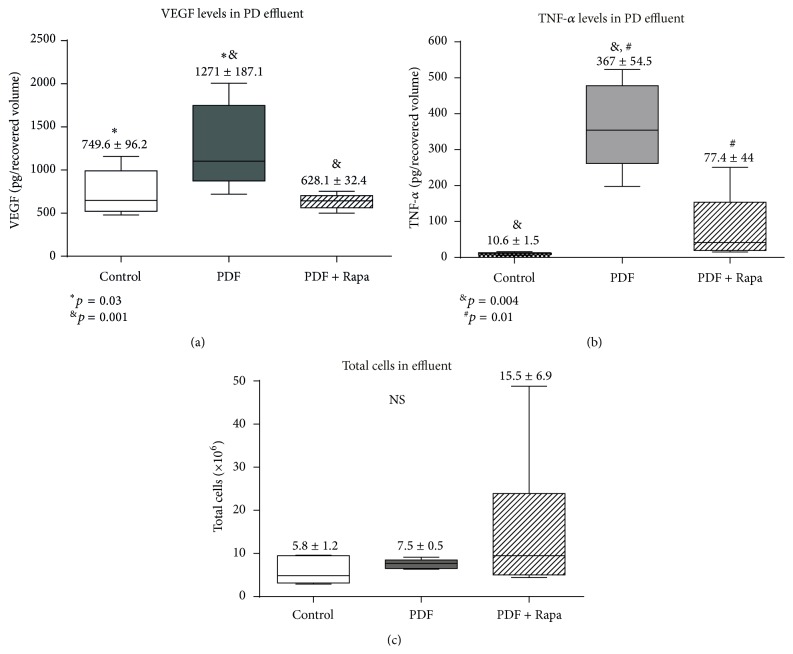
Rapamycin decreases VEGF and TNF-*α* levels in the peritoneal cavity. (a) Analysis of VEGF and (b) TNF-alpha in the drained volumes shows a strong increase of these factors in PD fluid-instilled animals, and administration of Rapamycin significantly reduces their production. The one-way ANOVA test resulted in a significance of *p* < 0.0038 and *p* < 0.0001, respectively. (c) Total numbers of cells in the drained effluent do not show statistical differences between groups. Box plots graphics represent 25th and 75th percentiles and median, minimum, and maximum values. Numbers above boxes depict means ± SE. Symbols represent the statistic differences between groups.

**Figure 5:**
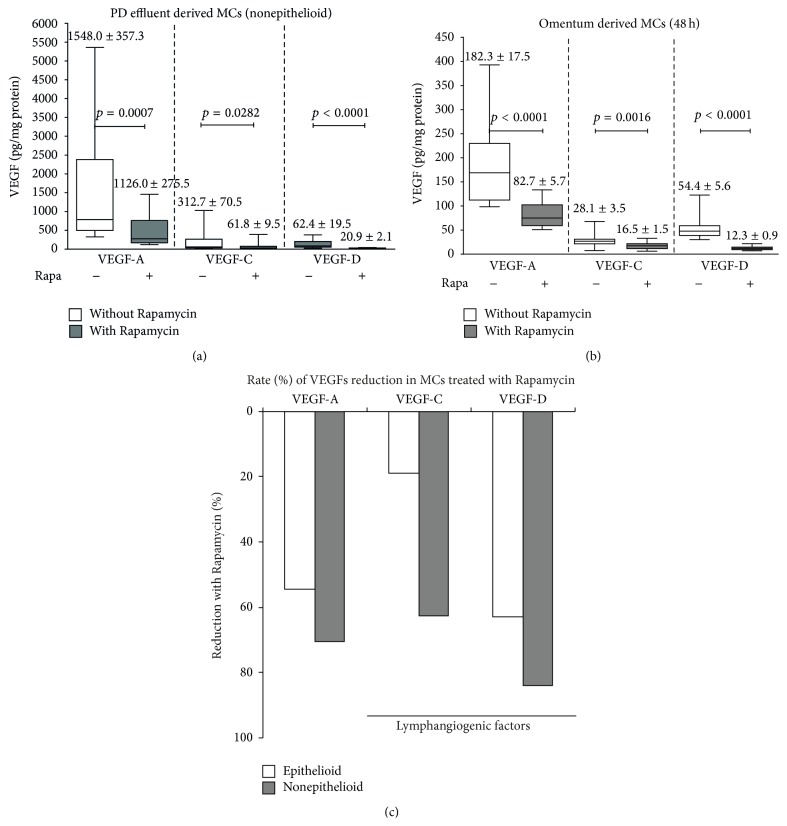
Rapamycin decreases the VEGF production by MCs. Omentum and PD effluent derived MCs were cultured and stimulated with Rapamycin 2 nM during 48 h. We performed two parallel cultures with equal numbers of MCs. A group received Rapamycin while the other was untreated. Supernatants were collected and VEGF-A, VEGF-C, and VEGF-D were measured. Rapamycin significantly decreased VEGFs, mainly the prolymphopenic VEGF-C and VEGF-D forms (a and b). We also calculated the reduction rate (%). Importantly in nonepithelioid MCs derived from PD effluent, VEGF-D was reduced by 82% and VEGF-C (gray bar graphic) by 63%. In omentum-derived MCs (white bar) the VEGF-D and VEGF-C were reduced by 63% and 20%, respectively (c). Box plots graphics represent 25th and 75th percentiles and median, minimum, and maximum values. Statistical differences between groups are shown (mean ± SD).

**Figure 6:**
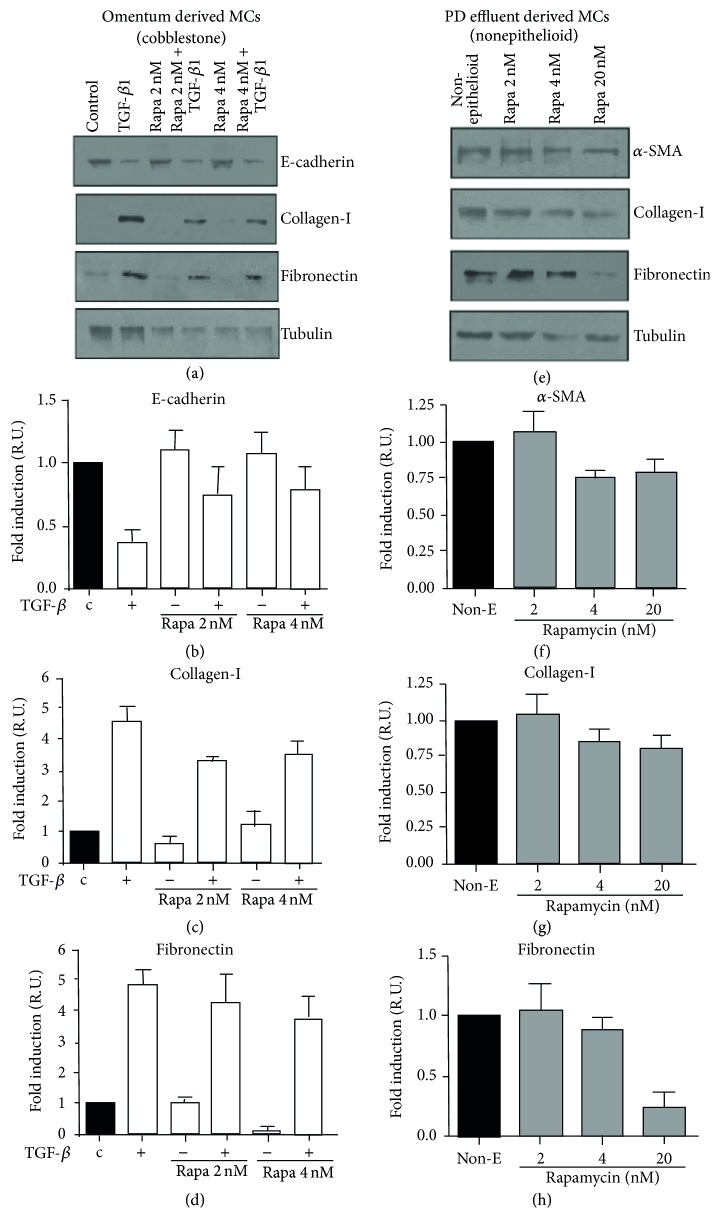
Rapamycin partially inhibits the MMT of MCs. (a–d) Omentum-derived MCs were treated or not with 1 ng/mL of TGF-*β*1 for 24 or 48 hours, in the presence of different doses of Rapamycin (2 and 4 nM). (a) Western blot analyses show that Rapamycin treatment prevents TGF-*β*1-induced E-cadherin downregulation as well as collagen I and fibronectin. (b) The E-cadherin expression was analyzed at 24 hours, whereas the expressions of (c) collagen-I and (d) fibronectin were analyzed at 48 hours of treatments. (e–h) Effect of Rapamycin on nonepithelioid phenotype MCs isolated from PD effluent (e) WB analysis in the presence of different Rapamycin's doses. Rapamycin inhibited the expression of (f) *α*-SMA, (g) Collagen-I, and (h) fibronectin expression in a dose-dependent manner (2, 4, and 20 nM). Results are presented relative to untreated MCs (black bars) which were arbitrarily assigned as value 1 (b to d and f to h). The experiments were repeated at least three times and results are depicted as means ± SE (bar graphics).

**Figure 7:**
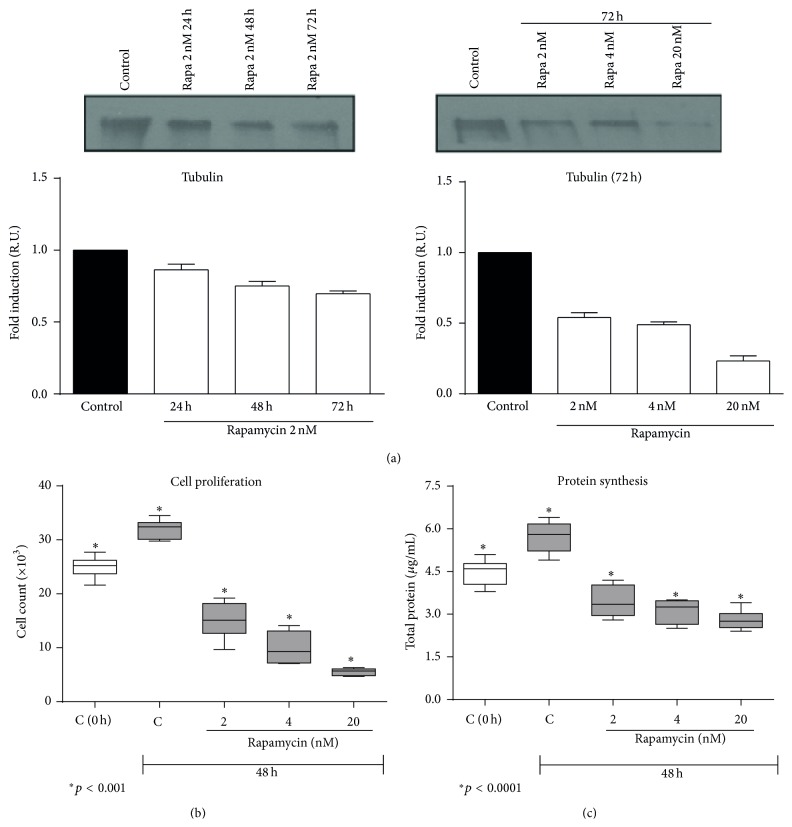
Rapamycin partially inhibited the MCs protein synthesis and MCs proliferation. (a) Rapamycin inhibits the tubulin expression in a time- and dose-dependent manner as shown in the Western blots analyses and their quantification graphics. Results are presented relative to untreated MCs (black bars) which were arbitrarily assigned as value 1. (b) MCs proliferation and (c) protein synthesis omentum cells cultured in semiconfluence. The first box represents baseline MCs (zero time). Second box shows the natural increases in MCs proliferation and protein synthesis at 48 h. The rest of the boxes show the decrease in both variables after the Rapamycin administration in a dose-dependent manner. In (b) and (c), one-way ANOVA analyses show a *p* value of *p* < 0.001 and *p* < 0.0001, respectively. Box plots represent the median, minimum, and maximum values, as well as the 25th and 75th percentiles.

Data from animal experiments and effluent parameters were compared with one-way ANOVA test and nonparametric Mann–Whitney rank sum *U* test (Figures 1–5).* In vitro* experiments were performed in triplicate. One representative WB picture was taken from each case and quantified using bar graphics (Figures 6 and 7). The cell proliferation and cell cycle experiments were repeated in five occasions (Figures 7(b) and 7(c)). We used the SPSS statistic package version 14.5 (Chicago, IL) and GraphPad Prism version 4.0 (La Jolla, CA). *p* < 0.05 was considered statistically significant. Box plot graphics represent the 25th and 75th percentiles and median, minimum, and maximum values (Figures 1–5 and 7).

## 3. Results

### 3.1. Rapamycin Ameliorated Peritoneal Membrane Thickening, Fibrosis, and MMT Induced by Dialysis Fluid Exposure in a Mouse PD Model

We analyzed whether Rapamycin, an mTOR inhibitor with recognized antifibrotic effect [12], might prevent the deterioration of the PM in a mouse model of PDF exposure. Histological analysis of parietal peritoneum biopsies from animals exposed to PDF (PDF group, *n* = 5) showed a loss of MCs monolayer and increased PM thickness when compared to control mice, which preserved the MCs monolayer (control group, *n* = 6). Oral administration of Rapamycin (2 mg/kg/day) to PDF-treated mice (PDF + Rapamycin group, *n* = 6) significantly reduced the peritoneal thickness (Figure 1(a)). Then, we analyzed the contribution of MMT to the number of submesothelial fibroblasts and fibrosis during PD.  Figure 1(b) shows the immunofluorescence staining of cytokeratin (red) and FSP-1 (green) (counterstained with DAPI in blue). The PDF exposure-promoted MMT (cells coexpressing cytokeratin and FSP-1 with double positive staining, yellow) is reduced by Rapamycin administration. Importantly, PDF group shows many costained MCs (yellow) in superficial areas (arrows) suggesting that MMT occurs at early stages of MCs transdifferentiation before invading the submesothelial area. PM thickness (*μ*m) increased in PDF group compared with control mice and PDF + Rapamycin group.  Figure 1(c) shows the statistic differences between the groups after a double blind count of PM thickness in ten arbitrarily chosen fields of optical microscopy. Since TGF-*β* is the major inducer of MMT and tissue fibrosis, we measured its levels in PD effluent. We found statistically significant differences between control group and PDF group, which showed maximum levels. Rapamycin group showed low TGF-*β*, which did not reach statistical significance (Figure 1(d)).  Figure 1(e) shows the count of MCs, which have suffered MMT. Given that PM thickness usually runs parallel to the MMT, Rapamycin showed less transdifferentiated MCs, as expected. These differences were statistically significant.

### 3.2. Rapamycin Decreased PD-Induced Angiogenesis and Endo-MT

Angiogenesis is an important process that occurs in the PM during PD [22], and a robust antiangiogenic effect of Rapamycin has been described in other tissues [12]. This drug acts by decreasing VEGF production or blocking its receptors [23]. To test the effect of Rapamycin on PDF-induced angiogenesis, blood vessels of the parietal peritoneum were stained with an anti-CD31 antibody. In the peritoneum from control mice, CD31 expression was confined to deeper vessels located in the muscular tissue (Figure 2(a)) while the PDF group showed a significant increase in the number of submesothelial vessels in comparison with PDF + Rapamycin group.

Although Endo-MT is a poorly known phenomenon in PD, it may contribute to PM fibrosis.  Figure 2(b) shows CD31 and FSP1 costaining in the submesothelial compact zone. Again, the PDF group showed more costained cells (yellow, see arrows) than the other groups.  Figure 2(c) shows the statistically significant differences between the groups in angiogenesis rate (submesothelial CD31^+^ vessels) and Figure 2(d) shows the number of endothelial cells suffering Endo-MT (CD31^+^ and FSP1^+^). Rapamycin group showed less MCs transdifferentiation than PDF group but similar to controls.

### 3.3. Rapamycin Decreased PD-Induced Lymphangiogenesis and Improved Peritoneal Ultrafiltration Rate

Lymphangiogenesis is another anatomical change associated with type-I PM failure. In PD, this process is poorly studied and seems to be closely related to the peritoneal water transport. In renal cancer, Rapamycin has been able to inhibit lymphangiogenesis, thus delaying the progression of this neoformative process [16]. Herein, we explore the effect of Rapamycin on lymphatic vessels formation in a PD mice model.  Figure 3(a) shows the immunohistochemical staining of podoplanin, revealing an increase of diaphragmatic lymphatic vessels during PDF exposition. Podoplanin stains MCs, so we can see two MC layers, one in contact with the pleura and the other thickened, facing the peritoneal cavity (magnification 10x). Amplifying the picture, we can see in the PDF group a dramatic increase in lymphatic vessels number (arrows). The number of these lymphatic vessels in Rapamycin-treated mice was similar to controls (Figure 3(b)). At the end of the experiment, we measured the UF rate. The PDF group presented an altered UF capacity in comparison to controls or Rapamycin group. These differences were statistically significant (Figure 3(c)).

Finally, we found that the number of blood and lymphatics vessels at the end of the experiment showed a significant linear correlation (*r* ^2^ = 0.78, *p* < 0.001), suggesting that both phenomena are in parallel (data not shown).

### 3.4. Rapamycin Decreased VEGF and TNF-*α* Levels in PD Effluent of PD

Treatment with Rapamycin decreased VEGF in the mice PD effluent (Figure 4(a)). This is consistent with our previous results showing that Rapamycin decreased angiogenesis and lymphangiogenesis. We also measured the effect of Rapamycin on inflammatory markers in the peritoneal cavity. To assess the peritoneal inflammatory state, we measured proinflammatory cytokines levels and the number of total cells in the PD effluent. Our results indicated that Rapamycin showed statistically significant lower amounts of TNF-*α* than that of the PDF group. Although we did not find significant differences in the total cells number between the groups (Figures 4(b) and 4(c)), an anti-inflammatory effect may be defended.

### 3.5. Rapamycin Decreased VEGF Production by Human Peritoneal MCs, Mainly Those with Prolymphogenic Effects

In PD, angiogenesis and lymphangiogenesis are two crucial factors involved in peritoneal transport, which are generally parallel to peritoneal fibrosis [24, 25]. Rapamycin is considered an anti-VEGF agent; therefore we explored its effect on VEGF-A, VEGF-C, and VEGF-D production by human peritoneal MCs. We isolated and cultivated MCs from omentum and PD effluent which were stimulated with Rapamycin 2 nM during 48 h. Both groups showed and important decrease in VEGF-A, VEGF-C, and VEGF-D supernatant levels especially the last two which are considered as prolymphogenic forms (Figures 5(a) and 5(b)).  Figure 5(c) shows the same data expressed in rate of decrease (%) of VEGF-A, VEGF-C, and VEGF-D decrease.

### 3.6. Rapamycin Attenuated MMT in PD Effluent and Omentum Derived MCs

Recent studies suggest that Rapamycin can have total or partial effect on MMT [26, 27]. For this reason we design the present experiment. We isolated and cultivated MCs from omentum and PD effluent, which were stimulated during 48 h with Rapamycin and/or rTGF-*β* to induce MMT. MCs were lysed and proteins extracted for analysis by WB.

Omentum-derived MCs were cotreated with Rapamycin and TGF-*β*. These did not show E-cadherin repression and had the Fibronectin and Collagen-I upregulation avoided, features of the MMT process (Figure 6(a)). Bar graphics show the measurement of the different gene expressions (Figures 6(b)-6(c)).

Figures 6(e)–6(h) show effluent derived MCs exposed to Rapamycin during 48 h. Rapamycin decreased *α*-SMA, collagen-I, fibronectin, and tubulin expression, suggesting that this drug not only reduces ECM but also decreases cellular protein synthesis in a dose-dependent manner (Figure 6(e)).

### 3.7. Rapamycin Blocked Cellular Proliferation and Protein Synthesis and Delayed Wound Heading

Given that Rapamycin downregulates tubulin expression, we wondered if this is due to a decrease in cell proliferation, decrease in protein synthesis, or cell death by toxicity or apoptosis. First, we confirmed that the decrease in tubulin expression was time- and dose-dependent (Figure 7(a)).  Figure 7(b) shows the normal proliferation rate of MCs in subconfluence after 48 h in culture. This normal cell proliferation rate was blocked by Rapamycin, to levels even lower than those obtained in controls. A similar situation occurred with cell protein synthesis. When we analyzed the cell cycle (Table 1), Rapamycin increased the rate of MCs in resting (M1) and apoptosis (M4) and decreased MCs dividing DNA (M2). This effect also showed a dose-dependent pattern.

Supplementary Figure 2 in Supplementary Material available online at http://dx.doi.org/10.1155/2015/989560 shows the effect of time in culture and Rapamycin doses over cell viability. Both factors decreased MCs life rate, but, in the worst case, dead MCs rate never exceeded 10%.

Given these results, we decided to explore the effect of Rapamycin over wound healing (WH). To close a wound it is necessary to maintain an adequate rate of cell proliferation, cell migration, and reproduction. As expected, Rapamycin delays WH to 72 hours showing again dose-dependent pattern (Supplementary Figure 3).

## 4. Discussion

In PD, type-I PM failure is characterized by progressive peritoneal thickening, inflammation, angiogenesis, and lymphangiogenesis, which ends in severe peritoneal fibrosis and peritoneal transport failure of water and solutes, shortening PM usefulness [24, 28].

To prevent PM damage, the main arrangement adopted so far has been to improve PDF biocompatibility (neutral pH or low GDPs). The administration of steroids, Tamoxifen, and some immunosuppressive drugs has become popular [29]. After promising results in animal and* in vitro* studies, the use of Rosiglitazone, COX_2_ inhibitors (celecoxib), Statins, and anti-TGF-*β* molecules (among others) has been proposed [30, 31]. In many cases, the rationale for using these agents was MMT inhibition. MMT is responsible for a great number of submesothelial fibroblasts derived from transdifferentiated MCs, which perpetuate peritoneal fibrosis and also angiogenesis and lymphangiogenesis [3, 6], since these cells are active VEGF producers [10, 22]. As our results demonstrate, transdifferentiated MCs showed up to 10 times greater VEGF production than nontransdifferentiated MCs [6]. Clinically, it has been accepted that the composition of PDFs, with high content of glucose, GDPs, the osmolarity, low pH, and the formation of AGEs appear as the major inducers for VEGF release, directly or indirectly through the synthesis proinflammatory cytokines [2, 3, 24].

To our knowledge, few studies have considered VEGF as a therapeutic target for type-I PM failure preventing strategies. Rapamycin has traditionally been considered as an inhibitor of VEGF synthesis [12] and some successful clinical and animal experiences in treating EPS have been published [18, 32]. Herein, we present evidences for using Rapamycin as an antifibrotic and antiproliferative agent for blood and lymphatic vessels in PM failure.

Either after an acute (peritonitis) or chronic (long-term PDFs exposure) peritoneal aggression, a physiologic autocontrolled cellproliferation process occurs. However, in some cases this autocontrol is lost, leading to peritoneal fibrosis. The antifibrotic effect of mTOR inhibitors can be explained by a direct effect on cell cycle [33]. Rapamycin increases the rate of MCs in resting, apoptosis, and dead rate, and decreases MCs dividing DNA, as evidenced by our results (Table 1). Similarly, Xue et al. [23] demonstrated that Rapamycin binds the FK-binding protein 12, resulting in a complex that leads to phosphorylation and inactivation of p70S6 kinase, which normally has a stimulatory function in the production of ribosomal components necessary for protein synthesis and cell cycle, resulting in cell cycle arrest in G1 phase [33, 34].

Tulek et al. [35] demonstrated that, in pulmonary fibrosis, the antifibrotic effect of Rapamycin can be explained by an anti-inflammatory effect mediated by a decrease in IL-13 and platelet-derived growth factor- (PDGF-) A and TGF-*β*1 and an increase in interferon- (IFN-) *γ* levels in bronchoalveolar lavage fluid. Our results are in agreement with these findings, as Rapamycin decreased TNF-*α* and TGF-*β* in mice peritoneal effluent (Figure 4). We also found that Rapamycin treatment diminished the PM cellular infiltration.

Another anti-fibrotic pathway of Rapamycin is the partial or total inhibition of MMT. In normal conditions, TGF-*β*1 induces MMT through* Smads* dependent and independent pathways [36, 37]. Recently, Patel et al. [26] demonstrated that Rapamycin inhibited Smad-3 signalling, blocking MMT, and tissue peritoneal fibrosis in a PD mice model. During MMT, TGF-*β* activates mTORC1 (one of the two functionally distinct complexes in which mTOR is present). This results in an increase in protein synthesis, cell size, motility, and invasion. This translational regulation complements the Smad-dependent transcriptional regulation induced by TGF-*β*. The mTORC2 complex phosphorylates Akt, thus contributing to its activation, but its exact role in TGF-*β*-induced MMT remains to be discovered [38, 39].

Gao et al. [40] suggested that the protective mechanism of Rapamycin is Slug- and Akt/mTOR-dependent. Furthermore, the inhibition of cellular migration by metalloproteinase- (MMP-) 2 and MMP-9 blockade played a crucial role as antifibrotic and anti-MMT.

If we understand MMT as a physiological tissue repair process that needs an active MC cycle and active proliferation and migration, the effects of Rapamycin on both processes would delay tissue repair, but considering that in type-I PM failure peritoneal repair is uncontrolled and the process exerts an excessive proliferation, we could speculate that Rapamycin, used for short periods, could be a therapeutic alternative.

Another important anatomical change suffered by the PM in PD is submesothelial angiogenesis [6]. Although angiogenesis is supposed to participate in water peritoneal transport disorders, to our knowledge, it has never been considered as a therapeutic target itself. Our results indicate that Rapamycin inhibited the VEGF synthesis* in vitro* and* in vivo*. Consequently much less angiogenesis and lymphangiogenesis were found in the mice treated with PDF alone (Figure 2). In this group, peritoneal transport was altered compared to controls or Rapamycin-treated animals (Figure 3(c)). Another mechanism this drug has been related to is an inhibition of angiogenesis and fibrosis by HIF1*α* blockade [41]. However, we did not analyze this pathway in the present study.

Recent studies have shown that, in several fibrosing diseases, endothelial cells can also suffer a mesenchymal transdifferentiation (Endo-MT), generally commanded by similar signaling as MMT [8]. The importance of this process is given by its contribution to tissue fibrosis [42]. Herein, we found important evidence of submesothelial Endo-MT in PDF group, while in PDF + Rapamycin group this process was practically nonexistent (Figure 2).

Recently, Zhang et al. [43], using bleomycin to induce* in vitro* Endo-MT in HUVECs cells, observed mTOR activation, and Rapamycin reduced the rate of Endo-MT via Slug inhibition. Effectively, we observed preservation of E-cadherin and inhibition of Snail upregulation by Rapamycin, in MC cultures treated or not with TGF-*β*. Although in this study we only quantified Snail, both transcription factors (Snail and Slug) are intimately involved in the induction of the transdifferentiation process [42].

On the other hand our results indicate that Rapamycin inhibited the prolymphangiogenic VEGFs forms in MCs cultures. Lymphangiogenesis is a scarcely studied process in PD and its involvement in PD functional disorders has recently been recognized [25]. Generally, the lymphatic proliferation operates in parallel with fibrosis and MMT and its principal inductor is possibly TGF-*β*. TGF-*β* is activated in the peritoneal cavity by PDFs, AGEs, glucose, and proinflammatory cytokines [4] and is able to induce MMT, Endo-MT, fibrosis, and VEGF production (which induces blood and lymph vessels proliferation) [24]. In early stages of type-I PM failure, proliferation of both types of vessels could be responsible for hyperfiltration. In fact, in end-stages, when water and solute peritoneal transport fails, VEGF-C and podoplanin kept their overexpression in peritoneal tissue [25, 44].

Excessive lymphatic fluid drainage from the abdominal cavity can also raise concerns because of its potential role over macromolecule and isosmotic solutions reuptake, as well as convective reabsorption of solutes that were already cleared from plasma by diffusion [1, 45]. Our results show that the PDF group presented higher counts of blood and lymph vessels, which is normally associated with hyperfiltration. However, this group showed a lower UF rate (Figure 3(c)). This apparent contradiction is explained by the moment UF capacity was measured, because of the changing water transport speed during the dwell. This change is due to the functional alterations presented in the PM in the PDF-treated group (Supplementary Figure 1). Recently, Morelle et al. used fluorescent albumin and fluorescence spectroscopy to monitor the UF capacity of the PM in mice lacking the Aquaporin 1 (AQP1) (a water channel) [46]. Using this model they conclude that lymphatic absorption occur later, after 30 minutes of fluorescent marker intraperitoneal injection. However, this study was performed in mice with virgin peritoneum. With our data we cannot conclude that the maximum UF, which occurs early in the PDF group, could be caused by peritoneal lymphatic absorption; nevertheless, we can confirm that the anatomical changes (angiogenesis and fibrosis) in our mice after 30 days in PD play a key role in the peritoneal water transport.

Regarding the antilymphangiogenic mechanism of Rapamycin, we focused on the VEGFs forms levels and found that VEGF-C and VEGF-D decreased with Rapamycin treatment. Recently, Zheng et al. [11] demonstrated that normally the lymphatic proliferation starts with P13/AKT activation, which activates the mTOR pathway. The mTOR blockade (Rapamycin) acts through a dual effect, inhibiting this complex and interfering with VEGF-Receptor-3 and VEGF-C intracellular signaling [28, 47].

## 5. Conclusion

Rapamycin protected the PM through an antifibrotic and antiproliferative effect on blood and lymphatic vessels. It also inhibited Endo-MT and at least partially the MMT. Although this drug has been shown to delay the MCs proliferation, migration, cell cycle, and wound healing, by slowing peritoneal tissue repair, our results suggest that Rapamycin, given for short periods, could be beneficial to treat early stages of type-I PM failure.

## Supplementary Material

Supplementary figure 1. Effects of PD on water peritoneal transport in the PD mouse model.
Supplementary figure 2. Effect of Rapamycin on MCs survival.
Supplementary figure 3. Rapamycin slows wound healing of the MCs in culture.

## Figures and Tables

**Table 1:** Effect of Rapamycin on cell cycle on MC (%gated).

Cell cycle	Control	TGF-*β*	Rapa 2 nM	Rapa 4 nM	Rapa 20 nM
(M1)	75.77	76.34	78.97	79.74	79.98
(M2)	5.81	3.96	3.41	2.95	2.68
(M3)	15.01	16.19	13.93	13.36	13.22
(M4)	3.41	3.42	3.69	3.75	4.12
