# Supplementary figures and images for: Rapamycin Protects from Type-I Peritoneal Membrane Failure Inhibiting the Angiogenesis, Lymphangiogenesis, and Endo-MT
Source: Biomed Res Int. 2015 Nov 25;2015:989560. doi: 10.1155/2015/989560 (PMC4673327; doi:10.1155/2015/989560)

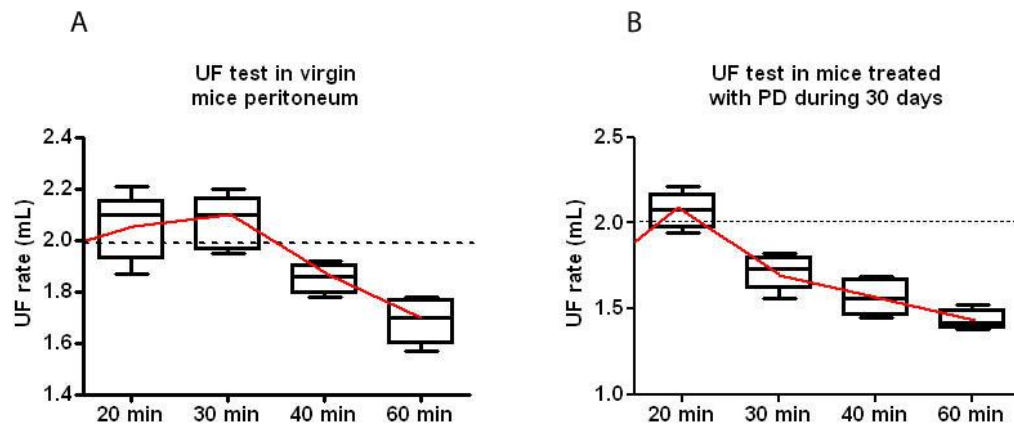

**Supplementary figure 1**

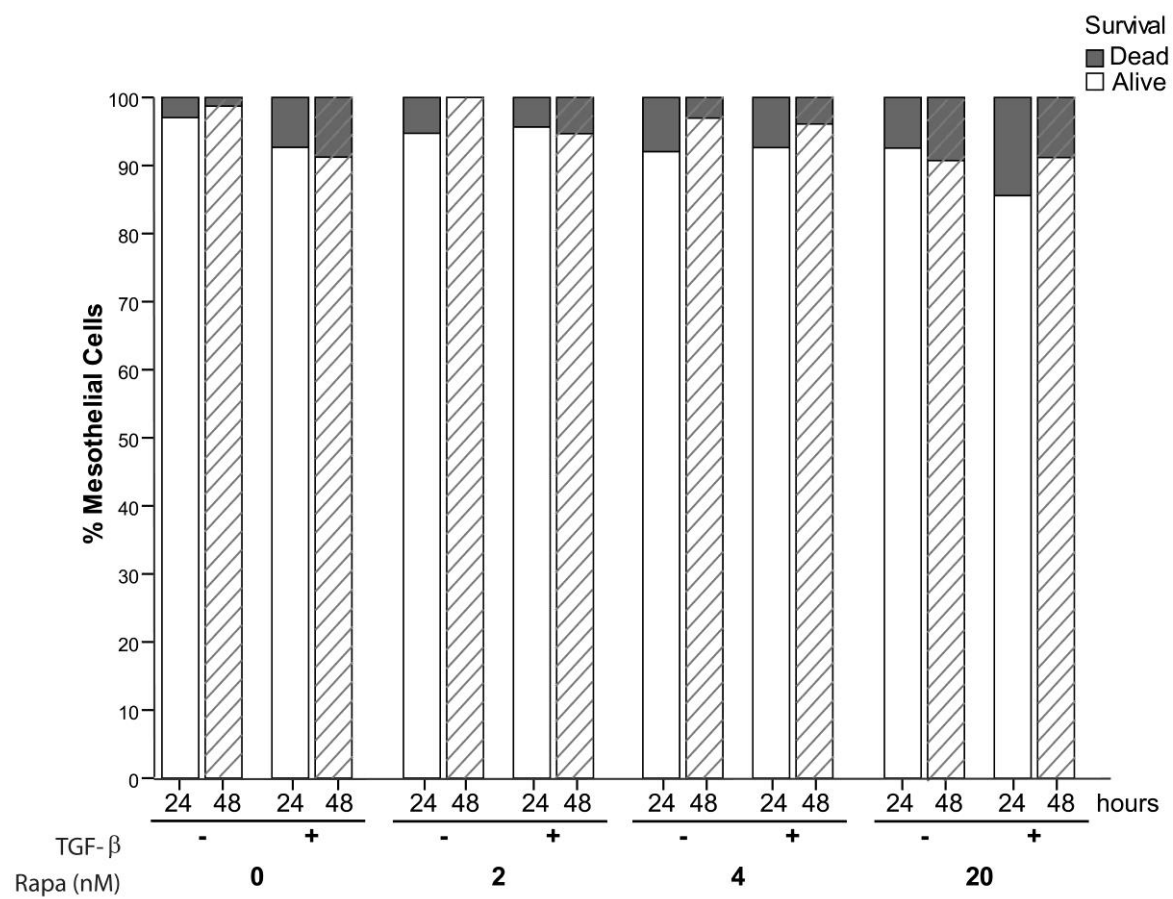

Supplementary figure 2

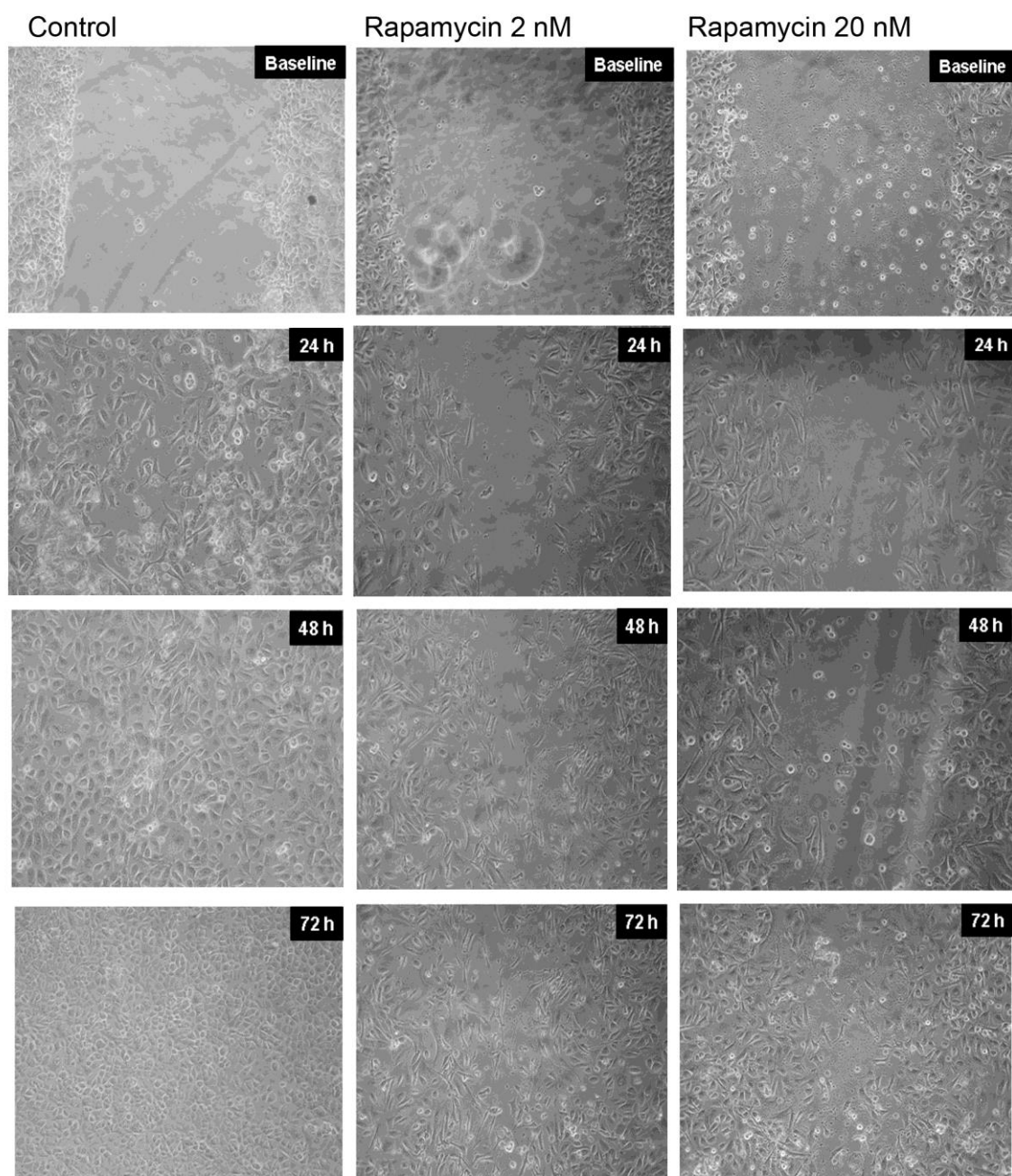

**Supplementary figure 3**

Supplement: Supplementary file 1 — Supplementary figure 1. Effects of PD on water peritoneal transport in the PD mouse model. Supplementary figure 2. Effect of Rapamycin on MCs survival. Supplementary figure 3. Rapamycin slows wound healing of the MCs in culture. [file 989560.f1.zip › 989560.suppl.pdf]
